# Supplementary material for: Complexome profiling of the Chlamydomonas psb28 mutant reveals TEF5 as an early PSII assembly factor
Source: Plant Cell. 2025 Mar 17;37(6):koaf055. doi: 10.1093/plcell/koaf055 (PMC12164590; doi:10.1093/plcell/koaf055)
Supplement: koaf055_Supplementary_Data [file koaf055_supplementary_data.zip › psb28_interactive.zip!/plots/intensity/qualityPlots/peptides/ALD6_Cre12.g501050.t1.2.html]

### Cre12.g501050.t1.2

Trivial name: ALD6  
SeqCoverage [median]: 8.40  
Mass: 62.35 kDa  
  
 

| Sequence | MissedCleavage | Mass [Da] | wtIntensity | muIntensity | Score |
| --- | --- | --- | --- | --- | --- |
| AADDPVLK | 0 | 827.44 | 440.57 | 0.00 | 75.11 |
| LRLDDQMLTLGR | 1 | 1429.77 | 0.00 | 2857.25 | 73.85 |
| NLYLDSAEDTR | 0 | 1295.60 | 804.58 | 2568.39 | 95.57 |
| SGLGVVGNPYLVDGVQK | 0 | 1700.91 | 518.68 | 1810.52 | 91.86 |
|  |  |  | 1763.8 | 7236.2 |  |
